# Analysis of FoxP3+ T-Regulatory Cells and CD8+T-Cells in Ovarian Carcinoma: Location and Tumor Infiltration Patterns Are Key Prognostic Markers

**DOI:** 10.1371/journal.pone.0111757

**Published:** 2014-11-03

**Authors:** Cecilia Hermans, David Anz, Jutta Engel, Thomas Kirchner, Stefan Endres, Doris Mayr

**Affiliations:** 1 Department of Pathology, Ludwig-Maximilians-University, Munich, Germany; 2 Tumorregister Munich, Institute of Medical Informatics, Biometry and Epidemiology, Ludwig-Maximilians-University, Munich, Germany; 3 Center of Integrated Protein Science Munich (CIPS-M) Division of Clinical Pharmacology, Ludwig-Maximilians-University, Munich, Germany; New York University, United States of America

## Abstract

**Purpose:**

Tumor infiltrating CD4+CD25+FoxP3+ regulatory immune cells (Treg) have been associated with impaired anti- tumor immune response and unfavorable prognosis for patients affected by ovarian carcinoma, whereas CD8+ T-cells have been found to positively influence survival rates in a large panel of solid tumors. Recently, density, location and tumor infiltration patterns of the respective immune cell subtypes have been identified as key prognostic factors for different types of tumors.

**Patients and Methods:**

We stained 210 human ovarian carcinoma samples immunhistochemically for FoxP3 and CD8 to identify the impact different immune cell patterns have on generally accepted prognostic variables as well as on overall survival.

**Results:**

We found that FoxP3+ cells located within lymphoid aggregates surrounding the tumor were strongly associated with reduced survival time (P = 0.007). Central accumulation of CD8+ effector cells within the tumor bed shows a positive effect on survival (P = 0,001).

**Conclusion:**

The distribution pattern of immune cells within the tumor environment strongly influences prognosis and overall survival time of patients with ovarian carcinoma.

## Introduction

Ovarian cancer has the highest mortality rate of all female genital cancers. Over 200,000 women are diagnosed with primary ovarian cancer every year [1]–[4]. Most of these cancers are diagnosed at a late stage, over 75% of patients are staged FIGO III or IV at first appearance. Overall five-year survival rates range between devastating 25% and 49% [2], [5]–[8]. Standard therapy, cytoreductive surgery followed by platin and taxan chemotherapy, initially leads to good response rates; however, in over 50% of the cases the disease recurs within the following five years [9], [10]. Generally accepted prognostic factors are optimal surgical debulking, histological subtype, tumor grading and staging [2], [6]. Nonetheless, these factors fail to predict overall survival rates accurately, since patients with similar clinical and pathological characteristics often differ widely concerning actual outcome and survival.

The correlation between tumor-infiltrating lymphocytes and prognosis of cancer patients has been investigated by numerous papers throughout the past thirty years [11]–[13]. For ovarian carcinoma Zhang et al. demonstrated as early as 2003 that CD3+ lymphocytes influence the progression-free and overall survival rates of patients [9]. Studies have since aimed at identifying methods to further depict the different cells that are involved in the anti-tumor immune response. As a result, a subpopulation of T-regulatory cells (Treg Cells) has been identified that plays a crucial role in tumor-induced immune suppression [14], [15]. The vast majority of Tregs are characterized by expressing CD3, CD4, CD25, the glucocorticoid- induced tumor necrosis factor receptor family related gene (GITR), the cytotoxic T-lymphocyte antigen-4 (CTLA-4) and, most importantly, the transcription factor forkhead box P3 (FoxP3) [16]–[19].

Treg cells are able to modulate the anti- tumor response of CD8+ effector T-cells and are associated with poor prognosis in ovarian carcinoma [20]–[24]. Similar results have been reported of numerous other tumors, for example colon carcinoma [25] gastric cancer [26] metastatic melanoma [27]. Curiel at al. showed that human tumor Treg cells suppress tumor-specific T-cell immunity and contribute to tumor growth in vivo. Treg cells were associated with a high mortality risk and reduced survival for patients with ovarian carcinoma [15]. Other authors have focused on the influence other subsets of T- lymphocytes have on the tumor environment. High numbers of and intratumoral penetration by CD8+ effector T-cells were found to slow down tumor growth and have been identified as independent prognostic factors for serous ovarian carcinoma [22]. On a similar note, a high CD8+ effector T-cell-to-Treg cell ratio, rather than the absolute number of each, has been suggested as an independent predictor of survival for patients affected by ovarian carcinoma [14] as well as breast cancer [28].

Apart from the determination of the mere presence of a distinct cell type within the tumor environment, increasing evidence suggests that the exact location and tumor infiltration pattern need to be considered [23], [27], [29]. Other authors have claimed the lymphoid clusters surrounding the carcinomata to be the actual site of Treg cell activation. The accumulation of FoxP3+ cells within lymphoid cell clusters was associated with a significantly worse prognosis than samples with high numbers of Treg cells [30]–[32].

This study is based upon the assumption that FoxP3 is a valid Treg cell marker. However, there have been publications that have questioned this assumption in the past [33], [34]. There also have been several reports of FoxP3 expression through tumor cells themselves, which has been interpreted as a specific tumor escape mechanism [35]–[37]. Some evidence also suggests that FoxP3+ Treg cell subpopulations may also be involved in tumor suppressive functions [38], [39]. The interaction of FoxP3 and the transcription factor NFAT are subject to further investigation to ultimately understand FoxP3 function [40]. Moreover, other immune cells are known to be able to exert immunsuppresive mechanisms, but do not express FoxP3 [41], [42], [43]. However, the single legitimate Treg cell marker still remains to be discovered and for now FoxP3 remains to be the most established and understood Treg cell marker we know [17], [27], [44]–[47].

## Material and Methods

### Patient characteristics

210 tumor tissue samples were selected from the archives of the Institute of Pathology of the Ludwig-Maximilians University, Munich, Germany. All patients had undergone debulking surgery at the Department of Obstetrics and Gynecology, Maistrasse, University Clinic Munich, between 1989 and 2002. The patients were between 27 and 87 years of age at diagnosis, with a median age of 60.1. Tumors were graded according to the Silverberg grading system. Most tumors were graded class 3+4 (62.2%) and only very few class 1 (2.96%). Only samples classified as stage III cancers, according to the International Federation of Gynecology and Obstetrics (FIGO), were included. All histological types of epithelial ovarian carcinoma were selected, but the serous (77.1%), endometroid (8.1%) and adenocarcinoma NOS (5.3%) were by far the most commonly represented types (Table 1).

**Table 1:** Patient and tumor characteristics.

	n
**Number of Samples**	210
**Age** (Mean/Range)	60.77(27–87)
**Survival Time** (Median/Standard Deviation)	48.28 (46,27)
**Grading**	Result (%)
G1	6 (2.96%)
G2	70 (34.48%)
G3+ G4	127 (62.56%)
**Histological subtype**	
Serous	162 (77.1%)
Mucinous	3 (1.4%)
Endometroid	17 (8.1%)
Adenocarcinoma (NOS)	11 (5.3%)
Undifferentiated	7 (3.3%)
Unknown	10 (4.8%)
**Optimal Surgical Debulking**	
Radical operation	49 (23.3%)
Remaining tumor < = 2 cm	68 (32.4%)
Remaining tumor >2 cm	43 (20.5%)
Unknown	47 (22.4%)
Sample laparotomy	2 (1%)

### Histology and immunohistochemistry

The formalin-fixed, paraffin- bedded blocks were cut into approximately <2 µm thick slices and mounted on SuperFrost Plus microscope slides (Menzel Gläser, Braunschweig, Germany). After deparaffinization and rehydration, sections were immersed into Dako Target Retrieval solution (Dako North America Inc., Carpinteria, USA), pH 6, 1∶10, incubated at 97°C–99°C at 750 Watt for 2x 15 minutes, and allowed to cool to room temperature for 20 minutes. Endogenous peroxidase activity was blocked by 10-minute incubation in 7.5% hydrogen-peroxide solution (Hydroxen Peroxide Solution, Sigma Aldrich Co., Munich, Germany). Immunohistochemical staining for FoxP3 was performed according to standard procedure using MACH-3 mouse alkalic phosphatase polymer detection kit from Biocare Medical Systems (Concord, USA). The slides were incubated with monoclonal mouse antibody. Chromogen Red (Dako North Amerika Inc., Carpinteria, USA) was used as chromogen for FoxP3 staining, and lastly hematoxylin counterstaining was done (Vector Laboratories, Burlingam, USA). An independent set of slides was selected to perform immunhistochemical staining for CD8+ T-cells. These specimens were automatically stained using Ventana Benchmark XT according to standard operating procedure with monoclonal mouse antibody (Anti- Human CD8- AB-1 Antigen, Vision, Fremont, USA). Additional single serial staining was performed for CD4 and CD20 automatically using Ventana Benchmark XT with monoclonal mouse antibody according to the XT ultraView DAB procedure (Table 2).

**Table 2:** Antibodys used in this study.

Antibody	Type	Dilution	IncubationTime	Source
Anti- Human FoxP3(Catalog Number ab20034)	Monoclonal; Mouse(Klon 236A/E7)	1∶180	60 min	Biocare medical systems,Abcam, Cambridge, UK
Anti-Human CD8(Catalog Number MS 457)	Monoclonal; Mouse(Klon C8\144 B)	1∶50	24 min	LAB Vision, Fremont, USA
Anti-Human CD20(Catalog Number M0755)	Monoclonal;Mouse (Klon L26)	1∶400		DAKO North America Inc.,Carpinteria, USA
Anti-Human CD4(Catalog Number NCL-CD4-368)	Monoclonal;Mouse (Klon 4B12)	1∶10		Leica Biosystems,Nussloch, Germany

Cells were first quantified by counting absolute numbers of 100 power fields per specimen (two independent investigators). After adequate group boundaries were defined, all remaining 110 specimens, as well as the initial 100 samples, were reevaluated semi-quantitatively. At least 35–40 high power fields were counted at 20- or 40-fold magnification (Axiovert Inverse Microscope, Carl Zeiss, Jena, Germany). The amount of cells was categorized according to a specific scoring system (Table 3).

**Table 3:** Scoring System.

	Group1	Group2	Group3	Group4
CD8	0–4	5–14	15–24	>25
FoxP3	0–4	5–14	15–24	>25

Preferred cell location (central vs peripheral) and finally, the infiltration of immune cells into the tumor tissue were checked (no infiltration vs. infiltration). Per definition, only cells surrounded by at least three tumor cells were counted as infiltrating the tumor.

### Statistical analysis

Statistical analysis was done by Superior Performance Software Systems Statistics Software (SPSS). Correlations between clinicobiological data and the FoxP3+ and CD8+ cell content were determined using a χ^2^ test. Survival curves were plotted using the Kaplan-Meier method and compared using the log-rank test. Multivariate analyses of prognostic factors for survival were performed using a Cox Proportional Hazard Model.

### Ethics Statement

Following the requirement of an ethics statement for publication of our manuscript in your journal, we would like to declare that the presented data were the result of a retrospective analysis on anonymized biopsy material. The material was sampled for diagnostic purposes. At all times we acted in accordance with the legal requirements concerning confidential medical communication as well as the data protection act. Consequently, we were sure before starting our investigation in 2009 that consulting the Institutional Review Board of out University was not required.

## Results

### a) Amount, Location, Infiltration of CD8+ and FoxP3+

**Figure 1:**
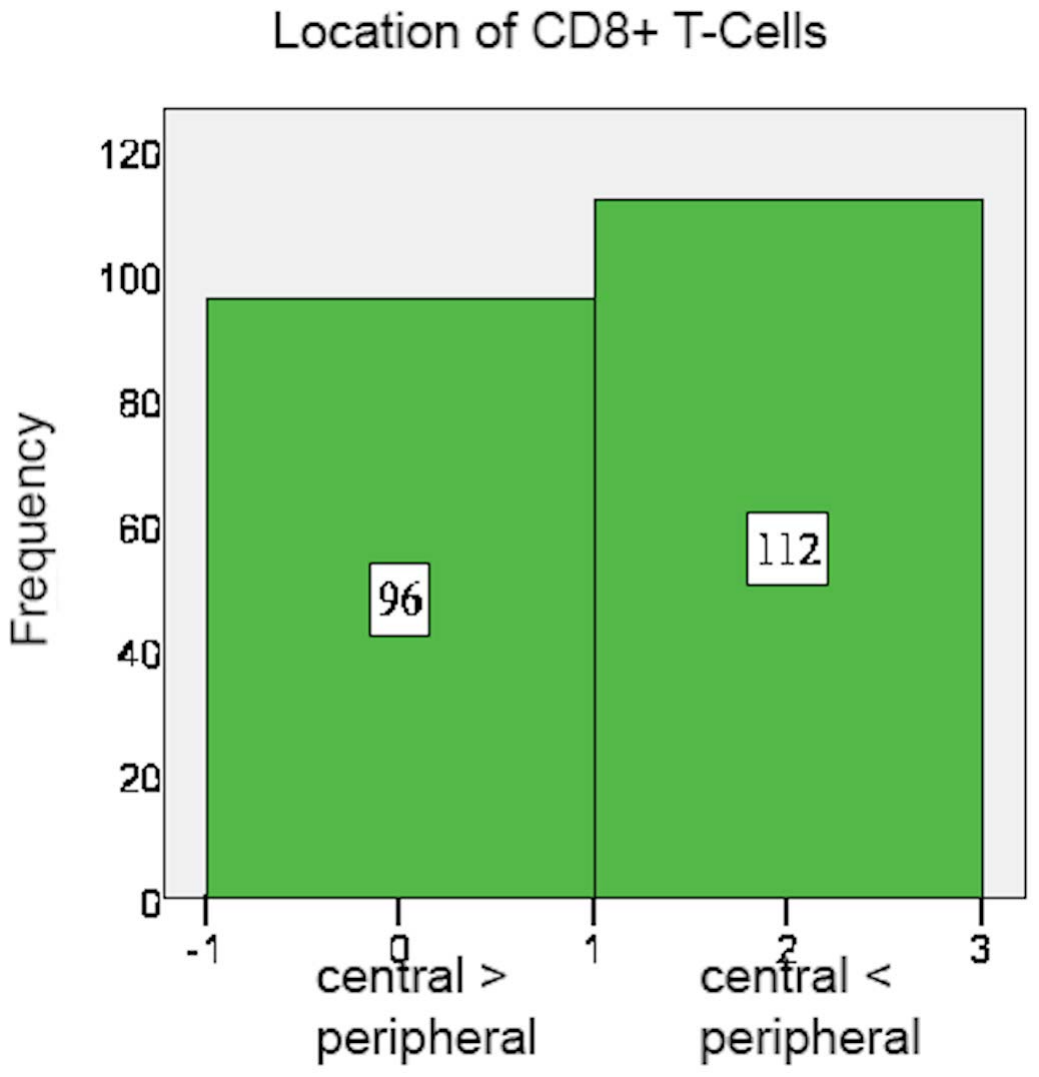
CD8+ T-cells: Location.

**Figure 2:**
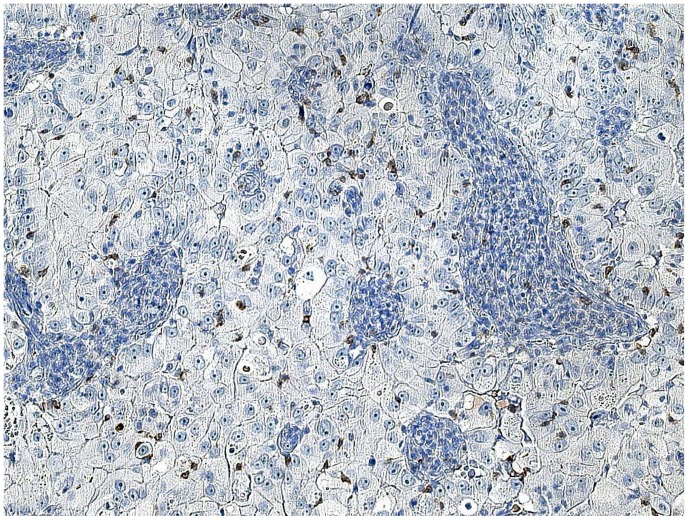
CD8+ T-cells infiltrating tumor tissue. Microscope: Carl Zeiss, Jena, Germany, 20-fold magnification, room temperature, Adobe Photoshop.

**Figure 3:**
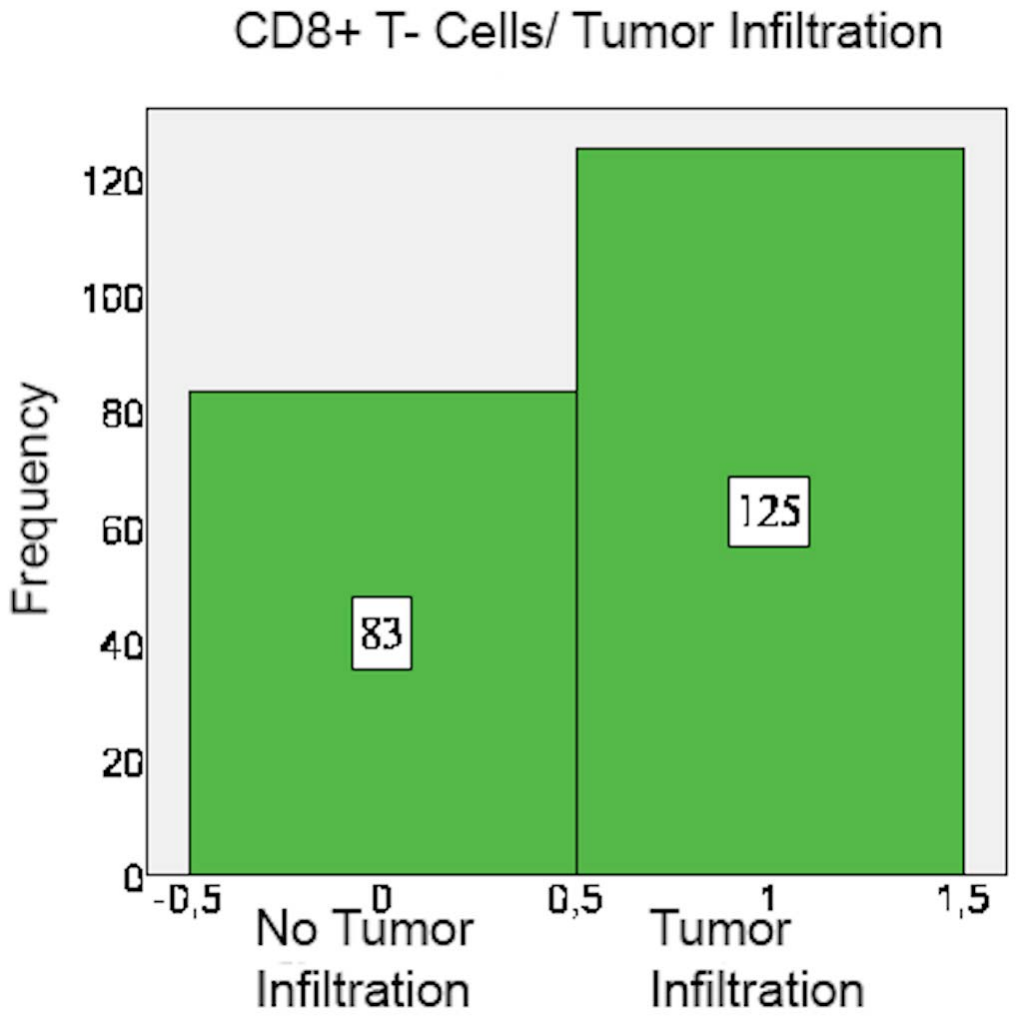
CD8+ T-cells: Tumor infiltration pattern.

CD8+ and FoxP3+ cells were detected in nearly all specimens, with CD8+ and FoxP3+ staining exclusively found on lymphocytes (Table 4). The amount of CD8+ cells mostly varied between 5 and 25 cells per field. In 53.8% of the examined specimens, CD8+ cells mainly accumulated in the peripheral stroma surrounding the tumor tissue and in 46.2% they spread equally throughout the tissue (Figure 1). A mere 60.1% of cases showed tumor-infiltrating CD8+ cells (Figures 2 and 3).

**Table 4:** Amount of cells.

	Group1	Group2	Group3	Group4	Missing
CD8	54 (25.7%)	78 (37.1%)	68 (32.4%)	8 (3.8%)	2 (1%)
FoxP3	65 (31.0%)	50 (23.8%)	59 (28.1%)	27 (12.9%)	9 (4.3%)

**Figure 4:**
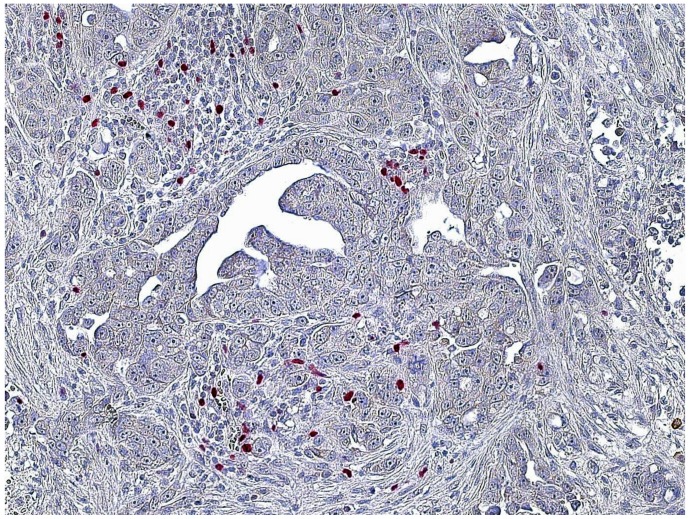
FoxP3+ cells mainly accumulate centrally. Carl Zeiss, Jena, Germany, 20-fold magnification, room temperature, Adobe Photoshop.

**Figure 5:**
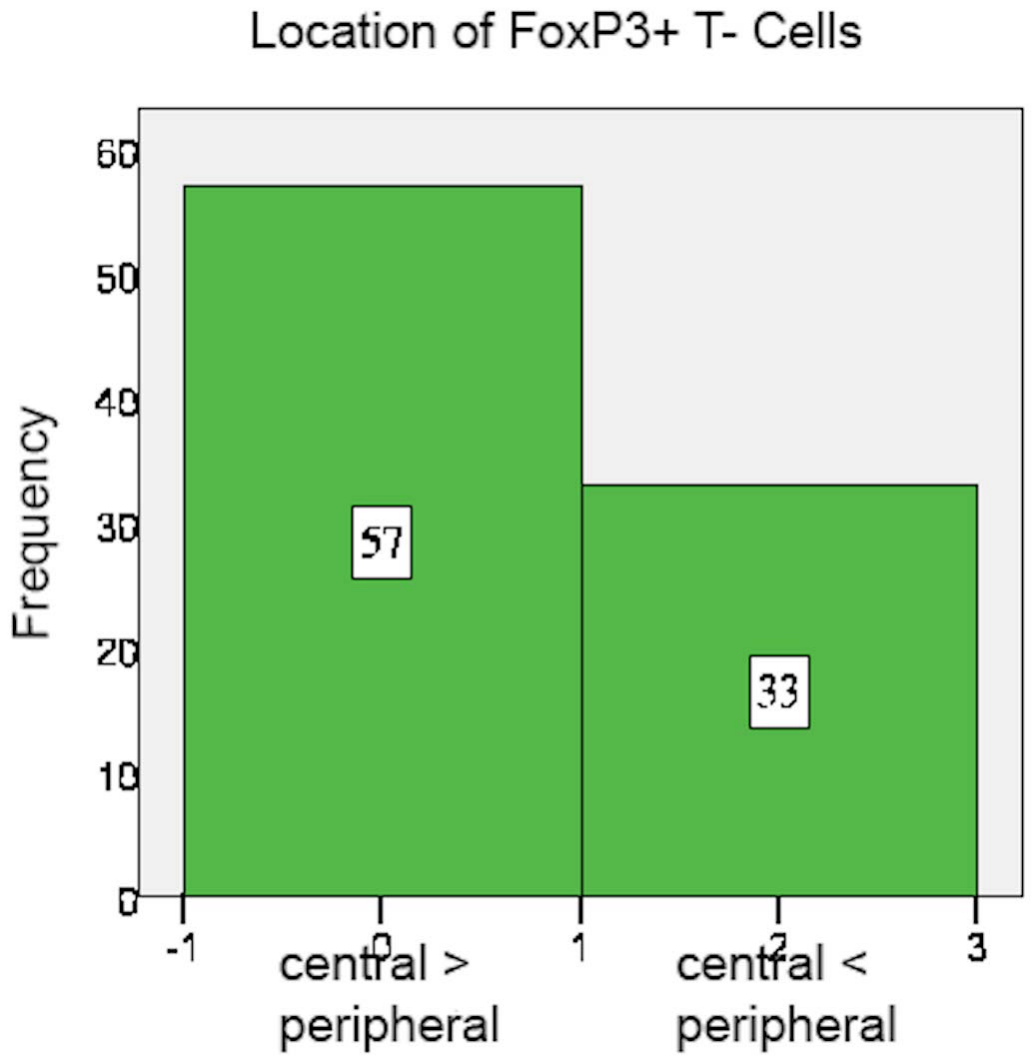
FoxP3+ cells: Location.

**Figure 6:**
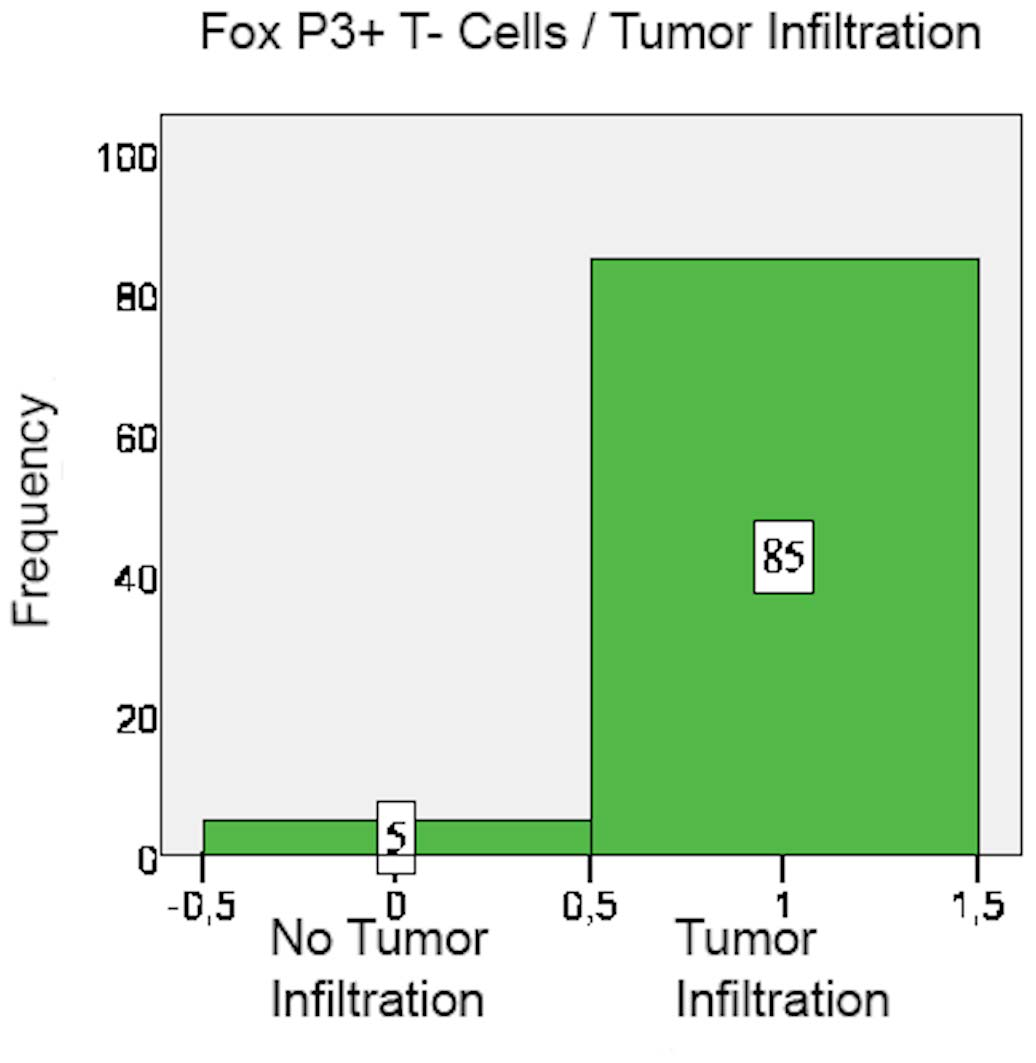
FoxP3+ cells: Tumor infiltration pattern.

FoxP3+ cell count mainly ranged between 5 and 25 cells per field as well, but overall more FoxP3+ than CD8+ cells were counted in the specimens. Moreover, FoxP3+ cells accumulated relatively more often within the centre of the specimen than in the peripheral stroma (63.3%) (Figures 4 and 5). FoxP3+ cells usually infiltrated the tumor tissue (94.4% positive tumor infiltration) (Figure 6) (Tables 5 and 6).

**Table 5:** Localisation of cells.

	Central> = Peripheral	Central<Peripheral	Missing
CD8	96 (45.7%)	112 (53.3%)	2 (1%)
FoxP3	57 (27.1%)	33 (15.7%)	120 (57.1%)

**Table 6:** Tumor infiltration of cells.

	Tumor not Infiltrated	Tumor infiltrated	Missing
CD8	83 (39.9%)	125 (59.5%)	2 (1%)
FoxP3	5 (2.4%)	85 (40.5%)	120 (57.1%)

### b) Correlation analysis

The amount of CD8+ cells within the specimen strongly correlates with the amount of FoxP3+ cells (p = 0.0001). Also, high numbers of CD8+ T-cells are positively linked to tumor infiltration of CD8+ cells (p = 0.001).

Increased numbers of FoxP3+ cells are associated with tumor infiltration of FoxP3+ cells, thus implying a benchmark number of Treg cells necessary for invasion into the tumor tissue (p = 0.001). Moreover, the ratio of CD8+T-cells to FoxP3+ cells strongly correlates with FoxP3+ cell location: a high CD8-to-FoxP3 ratio is associated with peripheral location of FoxP3+ cells. They mainly accumulate in lymphoid clusters surrounding the tumor. A low CD8+ to-FoxP3+ ratio, however, is associated with tumor infiltration of FoxP3+ cells. As soon as FoxP3+ cells outweigh CD8+ cells, they will infiltrate into the tumor and spread throughout the specimen.

The correlation tables reveal a significant relationship between “grading” level and FoxP3+ cell density, implying that high levels of FoxP3+ Treg cells are associated with significantly worse grading according to the Silverberg grading classification scheme (p = 0.004). This relationship is further emphasized by the negative correlation found between the CD8-to-FoxP3+ cell ratio and grading. Relatively low levels of CD8+ effector cells in comparison to FoxP3+ Treg cells are significantly associated with lower histological tumor grading (p = 0.001). Surprisingly, the parameter of “location of FoxP3+ cells” individually influences survival time (p = 0.007). Peripheral accumulation of Treg cells in lymphoid clusters surrounding the tumor bed is consequently associated with significantly less overall survival time than central accumulation within the tumor bed.

In order to quantify the influence generally accepted prognostic factors have on the prognostic model, statistical analysis was also done for common prognostic variables. “Age” (p = 0.001), “grading” (p = 0.001) and “optimal surgical debulking” (p = 0.007) showed significant effects on survival time, while neither the “employed neo-adjuvant therapy” nor “histological subtype” did. The lack of influence the parameter “histological subtype” shows is probably due to the lack of homogeneity of histological subtypes within the population.

### c) Cox Proportional Hazard Model

Using the Cox Proportional Hazard Model eliminated the statistical effects of the prognostic factors age, grading and surgical debulking. Three major individual prognostic factors for patients with ovarian carcinoma were then revealed. Firstly, the location of CD8+ T-cells within the specimen is an individual prognostic factor for patients with ovarian carcinoma (p = 0.001). Centralized accumulation of CD8+ effector cells in the tumor bed leads to prolonged survival time. Secondly, peripheral accumulation of FoxP3+ Treg cells in lymphoid clusters is a strongly significant negative prognostic factor for patients suffering from ovarian carcinoma (Table 7).

**Table 7:** Biomarkers and their influence on overall survival.

Biomarkers	No. Positive (%)	Overall survival (P)	Morphological pattern
**CD8+ cells**			
central	46.2	0.049	Central Location of CD8+ cells increases survival
peripheral	53.8	NS	
**FoxP3+ cells**			
central	63.3	NS	Peripheral Location of FoxP3+ cells reduces survival
peripheral	36.7	0.056	

NS: Not significant.

### d) Kaplan-Meier Curves and Log-Rank Test

**Figure 7:**
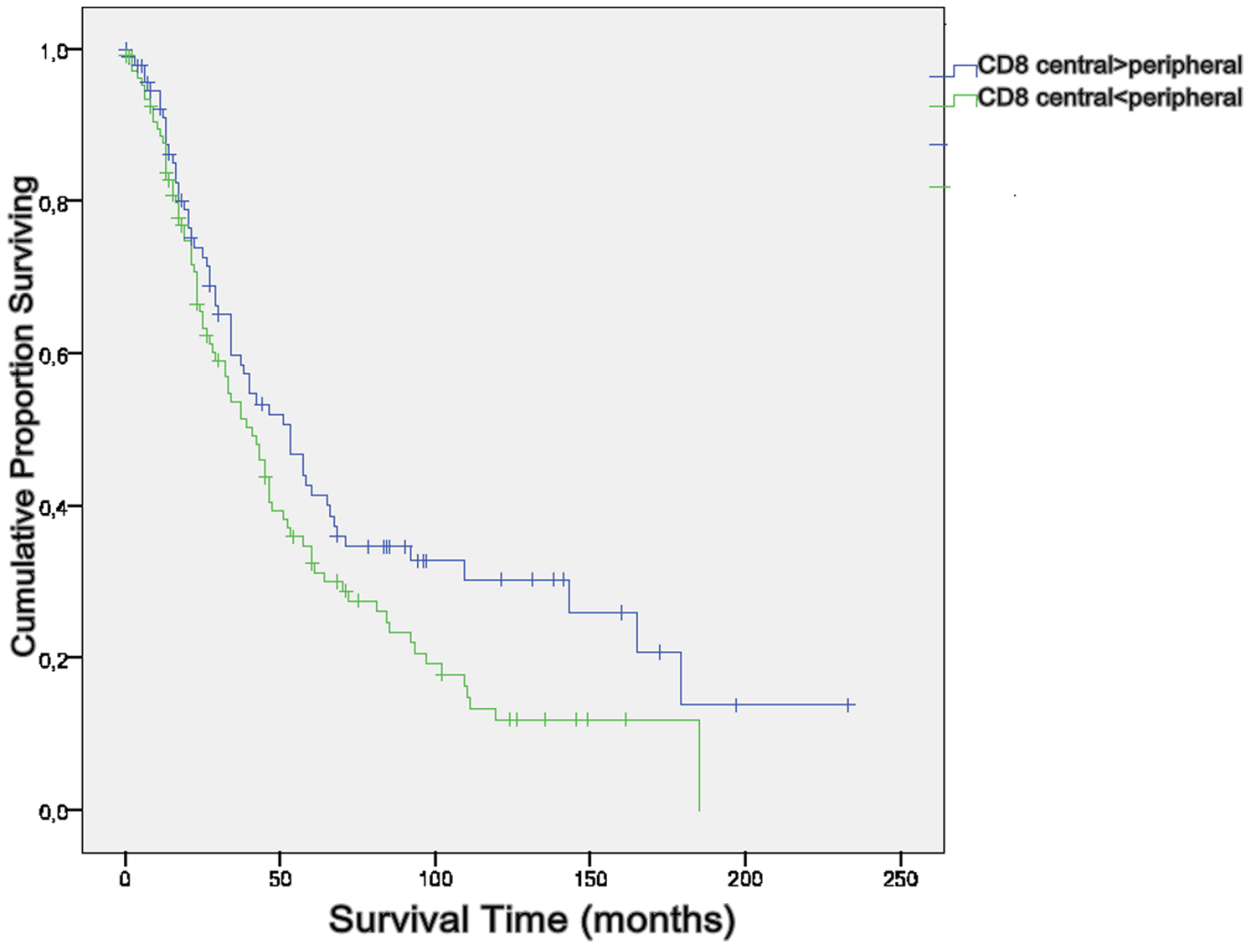
Log-Rank-Test CD8+. Central accumulation of CD8+ T cells is significally associated with survival (p = 0.049).

**Figure 8:**
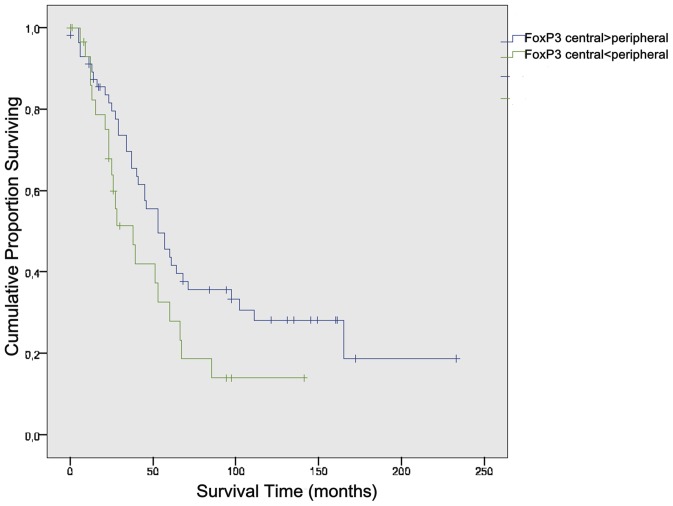
Log-Rank-Test FoxP3+. Peripheral accumulation of FoxP3+ Cells leads to reduced survival (p = 0.056).

The significant influence the location of CD8+ T effector cells as well as the location of FoxP3+ Treg cells within the specimen have on the overall survival time of a patient was reinforced by using the Kaplan-Meier Model (p = 0.049 and p = 0.056) (Figures 7 and 8).

## Discussion

This study examined the tissue of 210 patients with ovarian carcinoma for the infiltration of CD8+ effector T-cells and FoxP3+ T regulatory cells (Treg). In addition to the calculation of the respective frequencies, special attention was paid to the exact locations of the immune cells and to their capacity to infiltrate into the tumor bed. In order to adjust for known prognostic factors, multivariate analysis was done using the Cox Proportional Hazards Model introducing classical histophathological parameters such as grading, age at diagnosis, and surgical debulking success.

The current paradigm in tumor immunity suggests that tumor-associated antigens are captured by professional dendritic cells, which in turn prime naive T-cells to become antigen-specific anti tumor effector T-cells [48]. Ultimately, a large number of activated CD8+ effector T-cells should thus be able to attack the tumor tissue and eradicate the tumor. Unfortunately, as tumors actively fight to suppress effector cell function by producing immunosuppressive factors such as IL-10, TGFß, and VEGF, effective tumor eradication is rarely successful [49]–[52]. Moreover, the immunosuppressive function Treg cells exert on CD4+ and CD8+ T-cells have been identified as a crucial variable limiting an effective anti- tumor immune response. Yet, the mechanisms of suppression Treg cells make use of still remain to be fully understood.

The results of our study signify that the invasion of great numbers of CD8+ T effector cells is in fact one of the major prognostic factors for patients with ovarian carcinoma. This has previously been suggested for ovarian carcinoma as well as breast cancer by numerous authors [10], [14], [28], [53], but the focus of attention has so far not been placed on the location of the cells within the examined material, but on the mere amount of CD8+ T-cells and the ratio of CD8+ to other immune cells, respectively. The location, however, seems to be of greatest importance for an effective tumor attack. High numbers of CD8+ effector cells are also strongly associated with lower levels of histopathological grading, which strengthens the case that sufficient numbers of CD8+ T-cells within the tumor bed are actually able to keep tumor growth in check.

FoxP3+ Treg cells were mainly present within the tumor area, but less frequently they gathered in groups surrounding the tumor. In order to determine what cell types comprise these aggregates of immune cells we performed additional single serial staining for CD4 and CD20. We thereby could identify these peripheral immune cell aggregates as lymphoid structures. FoxP3+ expression in these lymphoid aggregates surrounding the tumor bed was found to be an independent prognostic factor for patients with ovarian carcinoma. Therefore, the presence of Treg cells within these lymphoid infiltrates is associated with a significantly higher risk of relapse and death. This finding has previously been reported for patients with breast carcinoma [31] as well as for follicular lymphoma [30]. Supposedly, this clinical observation results from the selective activation of Treg cells within these aggregates surrounding the tumor bed. Here, mature dendritic cells present tumor-specific antigens to peripheral naive FoxP3+ Treg cells, which then proliferate in situ and are thereafter capable of suppressing the anti tumor immune response of CD8+ effector T-cells through heterogeneous mechanisms including IL-10 and TGF-ß [32], [48], [51], [54]. This thesis was further strengthened by Gobert's finding that only Treg cells located in these lymphoid aggregates actually expressed activation markers such as HCA-DR, GITR and ICOS, while FoxP3+Treg cells located within the tumor tissue itself did not [31]. Previously, other authors have reported the expression of B7-H1 by dendritic cells within these aggregates, a receptor by which dendritic cells are able to prime the conversion of peripheral T-cells to functional Treg cells [48]. The accumulation of FoxP3+Treg cells within lymphocyte clusters surrounding the tumor, as well as abundant B7-H1 expression within this region, have lately also been reported for patients with prostate cancer [29]. Thus, it seems to be crucial not only to determine the density of FoxP3+ cells, but their exact location in the examined tissue. In order to influence the tumor microenvironment, the B7-H1 receptor seems to be one key mechanism which controls Treg cell activation.

On a different note but in accordance with the findings of this study, Jaffee et al. revealed that CD8+FoxP3+ cells are present in tumors only if there is an existing pool of tumor-rejecting effector CD8+ T-cells. Therefore, the accumulation and likely the tumor infiltration of FoxP3+ T-cells mark the presence of tumor-rejecting antigen-specific CD8+ T cells, which in turn serves as a marker for an effective T-cell response [55]. Similar results were presented by Nishikawa et al. as well as Rahir et al. [56], [57]. Overall, recent data is suggesting that a unique effector subset of FoxP3+ cells may exist which influences the tumor microenvironment in a favorable way and contributes to overall antitumor response. Given the multifaceted influence Tregs play in cancer progression, further research is urgently needed to develop targeted therapies.
